# Holistic processing of human body postures: evidence from the composite effect

**DOI:** 10.3389/fpsyg.2014.00618

**Published:** 2014-06-20

**Authors:** Sam Willems, Leia Vrancken, Filip Germeys, Karl Verfaillie

**Affiliations:** Laboratory of Experimental Psychology, Faculty of Psychology and Educational Sciences, University of Leuven (KU Leuven)Leuven, Belgium

**Keywords:** composite effect, body postures, holistic processing, body perception

## Abstract

The perception of socially relevant stimuli (e.g., faces and bodies) has received considerable attention in the vision science community. It is now widely accepted that human faces are processed holistically and not only analytically. One observation that has been taken as evidence for holistic face processing is the face composite effect: two identical top halves of a face tend to be perceived as being different when combined with different bottom halves. This supports the hypothesis that face processing proceeds holistically. Indeed, the interference effect disappears when the two face parts are misaligned (blocking holistic perception). In the present study, we investigated whether there is also a composite effect for the perception of body postures: are two identical body halves perceived as being in different poses when the irrelevant body halves differ from each other? Both a horizontal (i.e., top-bottom body halves; Experiment 1) and a vertical composite effect (i.e., left-right body halves; Experiment 2) were examined by means of a delayed matching-to-sample task. Results of both experiments indicate the existence of a body posture composite effect. This provides evidence for the hypothesis that body postures, as faces, are processed holistically.

## Introduction

Being able to adequately interpret socially relevant information on the basis of visual input is crucial for communication and interaction. Within the pool of socially significant stimuli, faces have been by far the most studied (e.g., Farah et al., 1998; Nelson, 2001; Webster and MacLeod, 2011; Piepers and Robbins, 2012; Rossion, 2013). However, like faces, body postures also contain social information and are visual stimuli to which human observers have developed a profound expertise (e.g., Zieber et al., 2010; Reed et al., 2012). In the present study, we focus on perceptual mechanisms involved in (human) body perception and possible (dis)similarities with face processing.

There is convincing evidence for the hypothesis that human faces are processed holistically (as well as analytically). It is not straightforward how to define exactly what constitutes a face feature (e.g., an eye region or even more fine-grained details like position of eye lids or color of eye irises), but we will adopt a relatively pragmatic approach by considering a feature as component stimulus information that is processed in a piecemeal and local manner (like an eye region). There is even less consensus in the literature on a precise definition of holistic, sometimes also referred to as configural face processing (e.g., Young et al., 1987; Searcy and Bartlett, 1996; Hole et al., 2002; Maurer et al., 2002; Bombari et al., 2009; Piepers and Robbins, 2012; Richler et al., 2012; we will use the term “holistic” in the remainder of the manuscript). Here, we start from the general working hypothesis proposed more than a century ago that a face is perceived as an undecomposed whole, rather than as a collection of individual features (Galton, 1883). More specifically, the (spatial) interdependencies between the local features of a face (e.g., a nose at a particular distance between two eyes and a mouth) or global configurations (i.e., a face being perceived as a unitary Gestalt with less information on subparts corresponding to individual features) are relatively more important than the facial features themselves (Carey and Diamond, 1977; Diamond and Carey, 1986; Tanaka and Sengco, 1997; Farah et al., 1998; Van Belle et al., 2010a,b).

**Figure 1:**
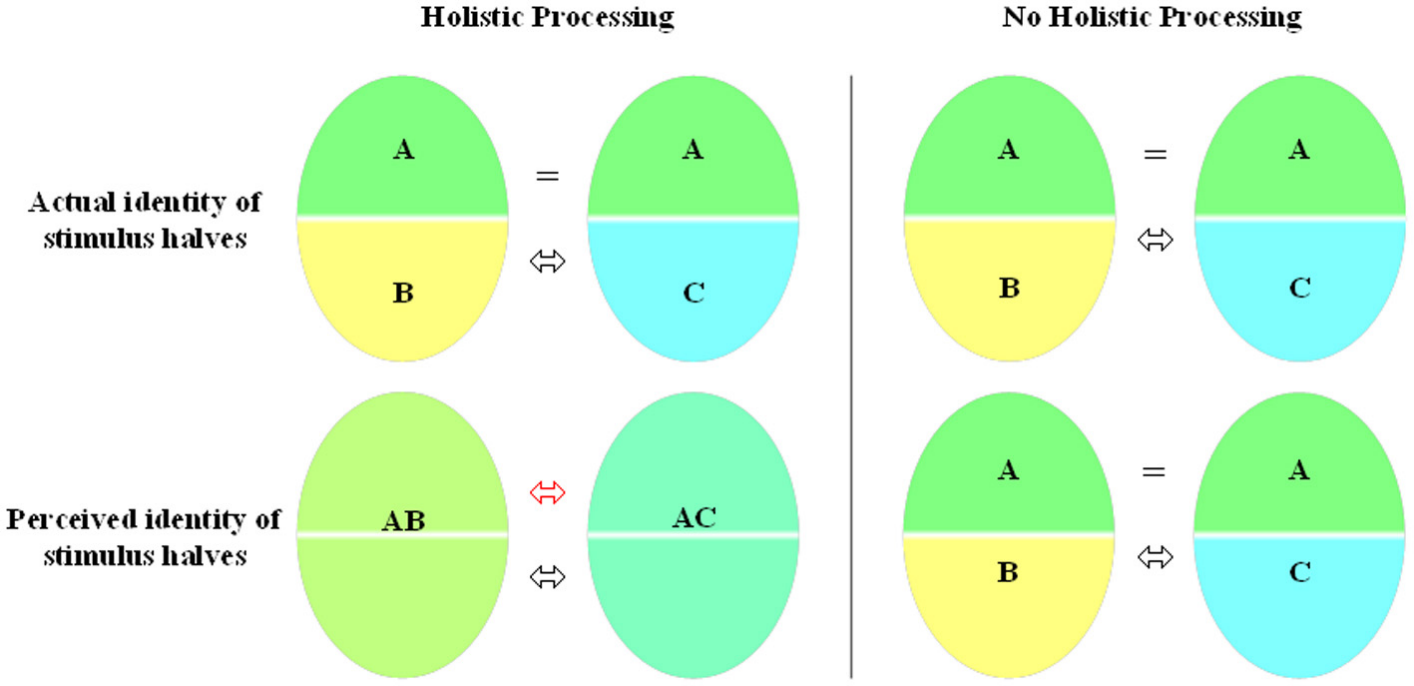
**Holistic processing explained within an identity-based composite paradigm.** In this example, participants have to judge the identity of the top part of the stimulus. Differential predictions can be made depending on the visual processing strategy that is involved in perceiving a stimulus class. On the one hand, when a stimulus class is processed holistically (left side), two identical top object halves will blend together with two different bottom halves making it hard for the viewer to perceive two identical top identities. On the other hand, when the information (i.e., identity) from the two halves is not integrated into a whole (right side), the viewer can effortlessly distract the two identities of a composite stimulus.

There are several lines of evidence supporting the hypothesis of holistic face processing (Verfaillie et al., 2014, provide a brief overview of a selection of this evidence). One effect that has become a gold standard in measuring holistic processing is the *face composite effect*. The effect was originally described by Young et al. (1987) for familiar faces and was later extrapolated to the recognition of unfamiliar faces by Hole (1994; Hole et al., 1999). It has since then been replicated and extended in several studies (e.g., Rossion and Boremanse, 2008; Rossion, 2013). In the now most frequently used delayed matching-to-sample version of the task (with unfamiliar faces), participants have to decide whether one half (mostly the top) of two sequentially presented faces is the same or not irrespective of the other face half (mostly the bottom). Interference is observed when two identical task-relevant top halves are combined with two different task-irrelevant bottom halves. When the bottom and top parts are spatially misaligned (i.e., shifted laterally), holistic processing is blocked and the composite face effect disappears, suggesting that, in the case of alignment of the two halves, the faces are processed as an integrated whole making selective attention to the top half more difficult (see Figure 1).

These findings paved the way toward the hypothesis that the composite effect allows researchers to qualitatively disentangle holistic processing from an analytic processing strategy and this for different kinds of stimuli. With inverted faces as stimuli, composite effects are absent or at least significantly moderated (Young et al., 1987; Robbins and McKone, 2007; Rossion and Boremanse, 2008). For several other object categories than faces, such as car fronts or dogs, no composite effects have been found (e.g., Gauthier et al., 1998; Robbins and McKone, 2007; Cassia et al., 2009; Soria-Bauser et al., 2011; Robbins and Coltheart, 2012).

For at least one object class other than faces there is evidence for holistic processing, namely human body postures. Indeed, just like for faces, both inversion effects (upright versions are processed in a qualitatively different way than inverted versions; Reed et al., 2003; Minnebusch et al., 2009, 2010) and part-whole effects [recognition of isolated body parts (arms, legs, or torso) is worse than recognition of body parts presented in the context of a whole body; Seitz (2002)] have been reported. The purpose of the present study is to examine a composite effect for body postures, with the aim to investigate whether body postures, like faces, are also perceived holistically. [Note that, although the present study does not provide direct evidence for this hypothesis, the suggestion of holistic body posture processing implicitly implies that we assume that, in the perception of body postures, a body schema, conceived of as a representation exclusively committed to the spatial relations between body parts is involved, e.g., indirect evidence for this hypothesis was provided by Daems and Verfaillie (1999), who documented long-term priming effects for anatomically possible body postures (presumably activating the body schema representation), but not for anatomically impossible postures and by Reed et al. (2003) who found inversion effects for possible postures but not for impossible postures; we are currently examining this issue further by comparing composite effects for anatomically possible and impossible postures.]

Recently, a delayed matching-to-sample protocol has been used to test for the presence of a body composite effect (Soria-Bauser et al., 2011; Robbins and Coltheart, 2012), but these studies provided mixed results. Soria-Bauser et al. (2011) investigated whether comparable composite effects can be observed for faces and bodies. They replicated the classical face composite effect, but failed to observe a similar effect for bodies and concluded that “holistic processing is not critically involved in the perception of human body forms” (Soria-Bauser et al., 2011, p. 201). Robbins and Coltheart (2012) did observe significant body composite effects, albeit that the effects were weaker than for faces and the vertical composite effect was more pronounced than the horizontal composite effect. At present, it is unclear why the results of the two studies diverge.

The investigations of Soria-Bauser et al. (2011) and Robbins and Coltheart (2012) share two (related) characteristics that set them apart from the present study. First, in the previous studies stimulus persons were always shown in a neutral upright standing posture with both legs straight on the ground surface and the two arms close to the body (either alongside the torso or crossed in front of the chest). Second, both studies used an identity-based approach toward body processing. This means that composites were created by combining two halves of different persons (e.g., the top half of person A is combined with the bottom half of person B) and the task of the participants consisted of deciding, on the basis of the task-relevant body part, whether two consecutively presented persons shared the same identity or not.

In the present study, we used a posture-based approach instead of an identity-based approach to the study of human body perception (Daems and Verfaillie, 1999; Reed et al., 2003, 2006; Ramm et al., 2010). In contrast to the studies of the body composite effect by Robbins and Coltheart (2012) and Soria-Bauser et al. (2011), stimuli consisted of a computer-generated model with the same identity on each trial but shown in different postures (not only neutral upright postures). More specifically, on each trial participants were presented with two body postures performed by the same figure and had to decide whether the predefined halves of the postures were the same or not. In Experiment 1, participants decided whether the upper body halves of two sequentially presented body stimuli had the same postural configuration regardless of the irrelevant bottom halves (horizontal composite effect). In Experiment 2, participants decided whether the right body halves of two sequentially presented body stimuli had the same postural configuration regardless of the irrelevant left halves (vertical composite effect). The decision to opt for the right body part as the task relevant part was arbitrary (the evidence for a right or left hemisphere processing advantage for body postures is somewhat mixed, e.g., Sokolov et al., 2012; Gilaie-Dotan et al., 2013).

In half of the trials, the task-relevant and task-irrelevant body parts were aligned (creating the possibility of holistic processing); in the other half of the trials the body parts were misaligned (blocking holistic processing). Robbins and Coltheart (2012) reported that the body composite effect based on identity was subtly stronger when body composites were created by combining a left half with a right half (i.e., vertical body composite effect) than when a top half was combined with a bottom half (i.e., horizontal body composite effect). As such, evidence suggests that left-right body integration is more prominent than top-bottom body integration. Vertical symmetry might play a prominent role here. Indeed, the human body can be defined as a torso with a head at apex and with arms and legs attached in a vertically symmetrical manner (Ramm et al., 2010). The importance of vertical symmetry of bodies has even been linked to mate choice (Robbins and Coltheart, 2012; note, however, that apparently this does not seem to hold to the same degree for faces where the horizontal composite effect is more pronounced than the vertical composite effect).

## Methods

### Participants

A total of 60 undergraduate students were recruited to participate in one of the two experiments. All participants were healthy subjects with normal or corrected-to-normal vision. Thirty subjects participated in Experiment 1 (*N* = 30; 25 females; *M* = 19.2 years, *SD* = 1.4), and another 30 participants took part in Experiment 2 (*N* = 30; 19 females; *M* = 20.2 years, *SD* = 2.8). All participants received course credit after completion of the experiment. Written informed consents were received from each participant conform to the guidelines of the Medical Ethics Committee of KU Leuven, Leuven, Belgium, and the ethical standards laid down in the 1964 Declaration of Helsinki.

### Stimuli and design

#### Experiment 1: horizontal body composite effect

**Figure 2:**
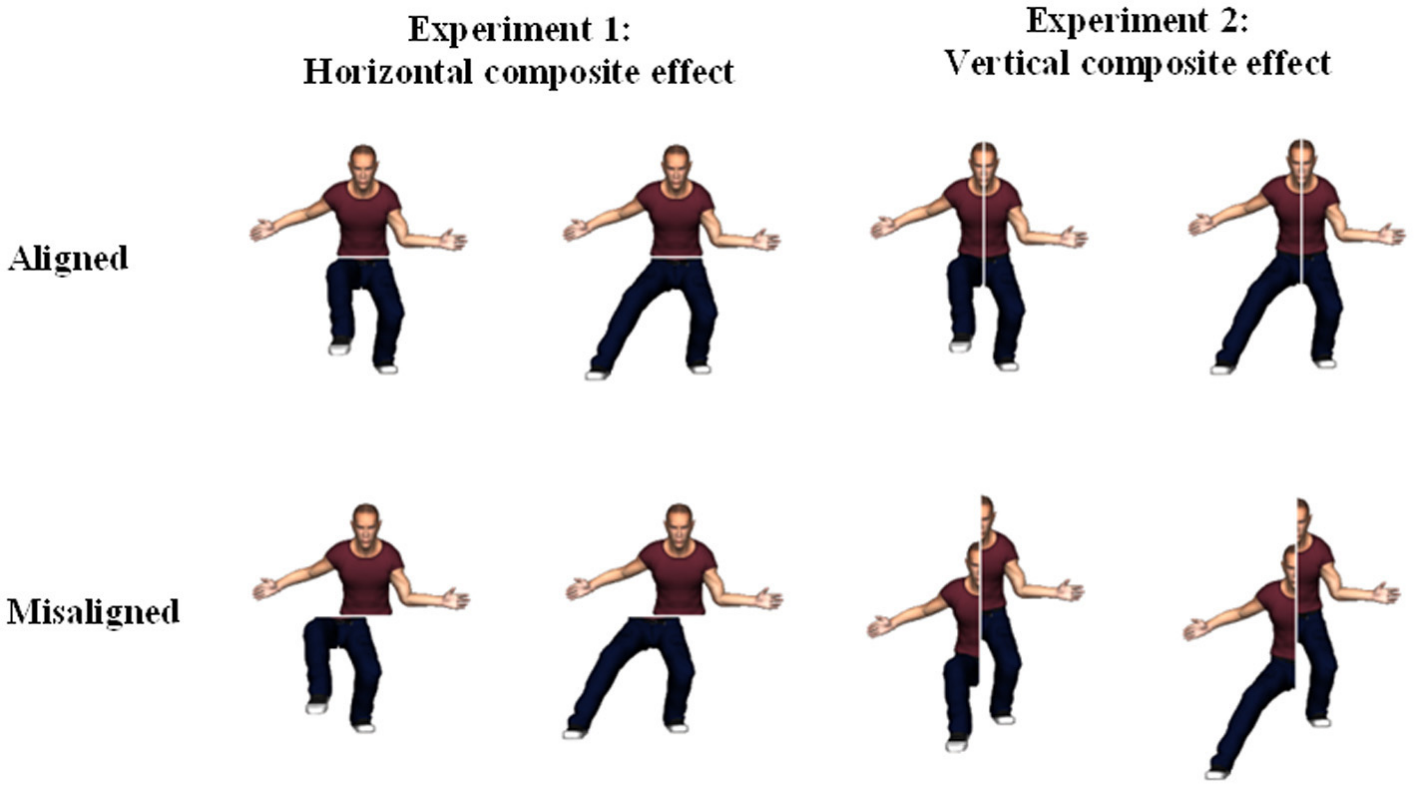
**Body composite stimulus pairs for each alignment condition for both experiments.** The relevant halves (i.e., top halves and right halves) in all four pairs are depicting an identical pose, while the irrelevant halves (i.e., bottom halves and left halves) differ within a stimulus pair.

Stimuli were created by using a male computer animated model (Poser 8, Smith Micro Software) with the posture-based approach in mind (see Figure 2). This approach means that a composite stimulus is composed out of body halves of the same model but possibly shown in different postures. On each trial a pair of consecutive body postures was shown. In Experiment 1, participants had to judge whether the upper halves (i.e., from the waist up) of the two stimuli within a pair were the same or not, irrespective of the lower halves (i.e., from the waist down). During stimulus construction, care was taken that the arms never crossed the boundary between upper and lower body. In all trials, the bottom parts of the stimuli in a pair were different (by changing the posture of one of the legs). The crucial condition to observe a composite effect is the condition in which the to-be-compared body halves (i.e., the task-relevant halves) remain the same. Twenty-six stimulus pairs were created where the upper body remained the same across the pair. Crucially, these 26 stimuli were either shown in an aligned version or in a misaligned version. Misaligned equivalents where created by horizontally translating the lower body from the upper body by approximately half the width of the torso of the model (~0.7°). The direction of this horizontal shift was counterbalanced. A small gap (width: 1.2 mm or ~0.1°) was added between the body halves in both the alignment and the misalignment condition. We also presented the mirror image versions of those stimuli, yielding a total of 104 stimulus pairs requiring a “same” response. In addition, we composed 9 stimulus pairs with different top and bottom halves. These stimuli were again presented in an aligned version or a misaligned version. Mirror images of these stimuli were also created, yielding a total of 36 stimulus pairs that required a “different” response. In total, each participant responded to 140 stimulus pairs. In addition, 12 stimulus pairs were created to be used in a practice run (an equal number of same top/aligned, different top/aligned, same top/misaligned, and different top/misaligned). [Note that we are using a partial design here. There is discussion in the literature (e.g., Richler and Gauthier, 2013; Rossion, 2013) whether this partial design actually is optimal or whether a complete design would be more appropriate. We are currently investigating this issue empirically in a new set of experiments on the body composite effect.]

#### Experiment 2: vertical body composite effect

In Experiment 2, the composite stimulus pairs of the first experiment were revised in order to examine left-right posture integration (see Figure 2). More specifically, the body stimuli were no longer segregated in a top and bottom half, but instead in a left and right half. The right side of the body from the viewpoint of the observer was chosen to be the task-relevant body half, while the left side took on the role of the task-irrelevant side (directions will henceforth always be defined from the perspective of the viewer). The right and left sides were defined by a vertical line (width: 1.2 mm or ~0.1°) through the middle of the head and body. The misaligned versions were generated by vertically translating the left side up- or downwards (counterbalanced) by one face length (~1.3°). Analogously to Experiment 1, there were 26 pairs with the same right body part but an aligned different left part and 26 pairs with the same right body part but a misaligned different left part (each shown in a mirror reflected version, yielding 104 trials that required a “same” response) and 9 pairs with a different right body part but an aligned different left part and 9 pairs with a different right body part but a misaligned different left part (each shown in a mirror reflected version, yielding 36 trials that required a “different” response). This resulted in 140 pairs of stimuli in total. There were again 12 practice trials.

### Procedure

**Figure 3:**
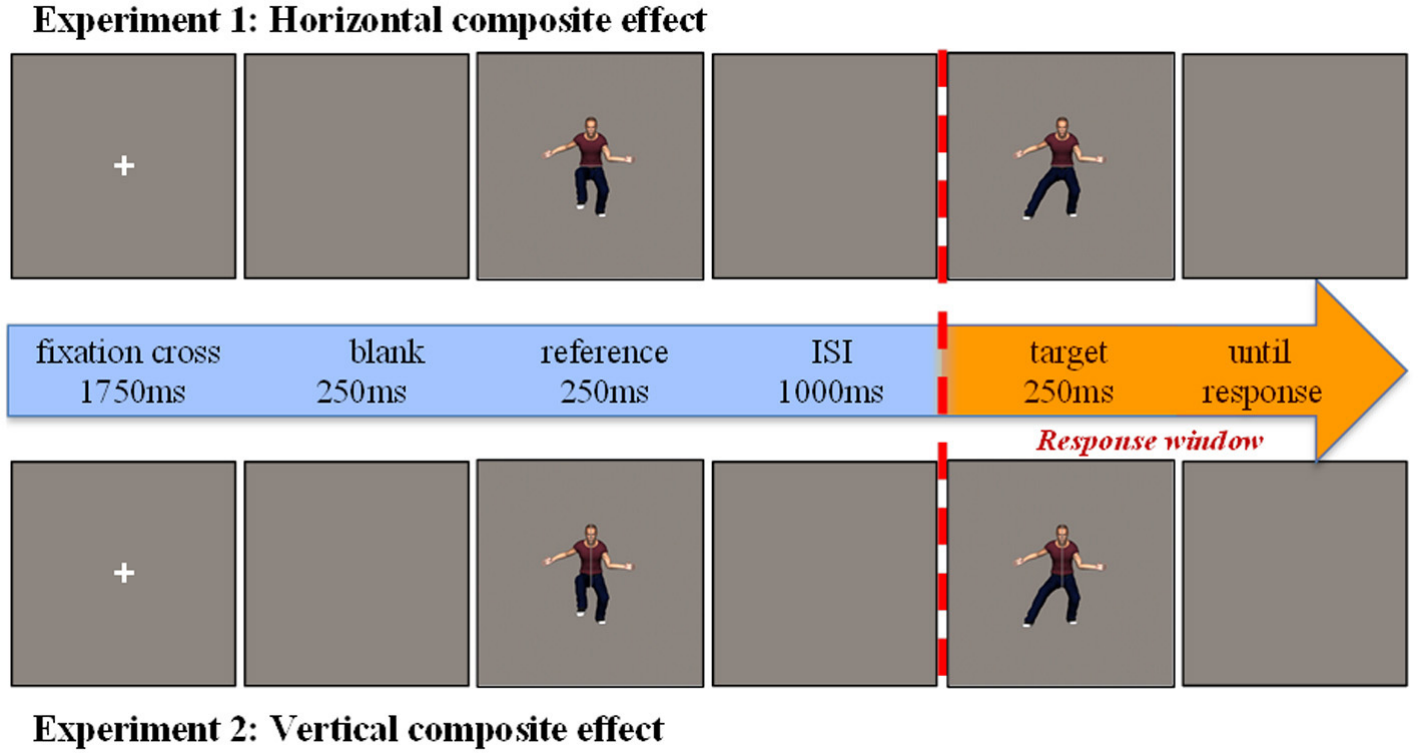
**Experimental procedure.** In both cases an aligned composite pair is shown in which the relevant body half (i.e., top half and right side, respectively for Experiments 1 and 2) is identical and the irrelevant half differs (i.e., bottom half and left side, respectively for Experiment 1 and Experiment 2).

In both experiments we used the same experimental procedure. All 140 stimulus pairs of one experiment were presented twice to the participant in four successive blocks of 70 trials each. Before the experimental procedure started, a practice run with 12 practice trials was presented to the participants. Stimulus pairs were shown in a modified delayed matching-to-sample task (see Figure 3). Each trial started with a fixation cross (0.5° × 0.5°) for 1750 ms centered on the screen, which was followed by a 250 ms blank screen. The two stimuli (on average: 4.3° × 14.8°) of a stimulus pair were presented 250 ms each in a sequential fashion in the center of the screen with a fixed interstimulus duration of 1 s. Participants were instructed to assess whether the upper (in Experiment 1) or the right (in Experiment 2) body halves of the sequentially presented stimulus pair were identical or not by pressing a green (*s*-key on keyboard) or red key (*l*-key on keyboard) respectively. Responses could be given from the target stimulus onset onwards. Although there was no response time cut-off defined, participants were instructed to give an answer as fast as possible without making unnecessary mistakes and thus warning subjects for the potential of a speed-accuracy trade-off. After giving a response in the practice run, a feedback screen was presented for 1000 ms. No feedback was given during the experimental procedure.

### Apparatus

Both experiments took place in a dimly lit room. Participants were seated in front of a 21-inch IIyama HM204DT CRT monitor with a predefined resolution of 1024 by 768 pixels at 75 Hz. During the practice and experimental procedure, subjects placed their chin on a chin rest at a fixed distance of 58 cm. The experiment was run on a Dell Precision 670 computer running Windows XP. Experimental control of stimulus presentation was delivered by Affect 4.0 (Spruyt et al., 2010) which recorded response times (in ms) and accuracy rates (0 or 1) per trial.

### Data analyses

Data analysis preparations included a thorough inspection of the data obtained from each subject for speed-accuracy trade-offs by looking at accuracy rates of the different trials. Subjects were omitted when they failed to reach a 80% accuracy rate on these trials. This resulted in the removal of two subjects (78 and 66%) in Experiment 1 (*n* = 28) and of four subjects (64, 78, 77, and 79%) in Experiment 2 (*n* = 26). Next, outliers were removed for each subject independently. Outliers were based on extreme reaction times with a different definition for lower-bound and higher-bound outliers due to a skewed distribution. Lower-bound outliers were defined as response times faster than 250 ms as these would not seem likely to be triggered by an actual perceptual response. Higher-bound outliers were defined as response times slower than three standard deviations above the subject's average. This resulted in the removal of less than 10 trials per subject.

From the remaining data, dependent variables were defined for each subject and condition. These included proportion correct responses, correct response times, and inverse efficiency scores (i.e., mean response time divided by the proportion correct responses or mean response time per correct response; Townsend and Ashby, 1978, 1983) as a compound index of accuracy and reaction time that takes any remaining speed-accuracy trade-offs into account. [There is discussion in the literature on the usefulness of inverse efficiency scores; see Bruyer and Brysbaert, 2011; Ramon and Rossion, 2012; note, however, that our data confirm to the criteria listed by Bruyer and Brysbaert (2011), in which case an analysis of inverse efficiency scores is useful; in addition, we do not replace the analysis of accuracy and RTs by an analysis of inverse efficiency scores; instead, we view it as a complementary analysis]. In line with previous studies (e.g., Soria-Bauser et al., 2011; de Heering et al., 2012; Robbins and Coltheart, 2012) and because of the used skewed trial distribution, data for same and different trials were analyzed independently from each other. Repeated measure ANOVAs were performed for each trial type with Alignment and Block number as within-subject factors, to test the presence of a composite effect and the stability of the effect throughout the experimental procedure, respectively. Alignment effects were also compared across the two experiments by using independent *t*-tests to test for differential alignment effects. When needed, sphericity violations were corrected by the Greenhouse-Geisser method. Partial eta squared values (${\hat{\eta }}_{p}^{2}$) are reported as a measure of effect size.

## Results

**Figure 4:**
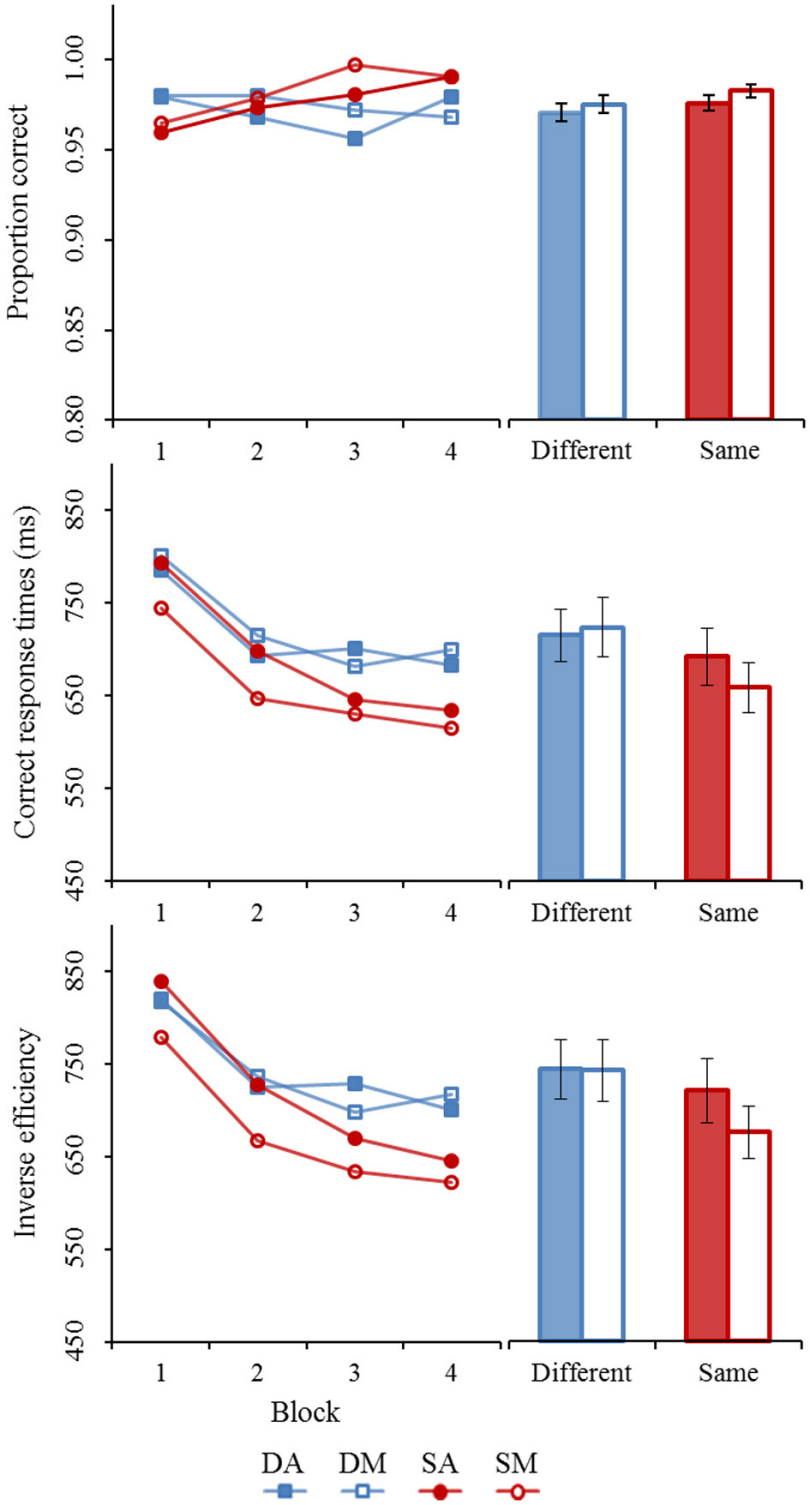
**Summary results of Experiment 1 (horizontal body composite effect).** Each panel shows the averages for each block level (1–4) and for each trial type (different vs. similar). Averages across the blocks are also shown. Error bars represent standard error of the mean. (DA, different aligned; DM, different misaligned; SA, same aligned; SM, different misaligned).

Results from Experiment 1 are visually summarized in Figure 4. The repeated measure ANOVAs revealed only a marginally significant accuracy-based composite effect within the same trials [Alignment: *F*_(1, 27)_ = 4.00, *p* < 0.06, ${\hat{\eta }}_{p}^{2}$ = 0.13] after taking the experimental progression into account [Block: $\hat{\varepsilon }$_GG_ = 0.67, *F*_(2.02, 54.44)_ = 9.57, *p* < 0.01, ${\hat{\eta }}_{p}^{2}$ = 0.26). The misaligned same trials (*mean* = 0.9827, *SE*_M_ = 0.0042) are only slightly better performed at than the aligned same trials (*mean* = 0.9758, *SE*_M_ = 0.0038), possibly due to a ceiling effect. Despite the small effect size, the accuracy-based composite effect was found to be stable throughout the experimental procedure [Alignment × Block: $\hat{\varepsilon }$_GG_ = 0.77, *F*_(2.31, 62.44)_ = 1.20, *p* > 0.10, ${\hat{\eta }}_{p}^{2}$ < 0.05]. Although the composite effect is based on a comparison of aligned and misaligned stimuli in the “same” trials, we also analyzed the “different” trials. As expected, results of the different trials did not provide evidence an alignment effect [*F*_(1, 27)_ = 0.34, *p* > 0.10, ${\hat{\eta }}_{p}^{2}$ < 0.05] after taking Block into account [$\hat{\varepsilon }$_GG_ = 0.93, *F*_(2.80, 75.64)_ = 0.67, *p* > 0.10, ${\hat{\eta }}_{p}^{2}$ < 0.05]. With the aligned (*mean* = 0.9706, *SE*_M_ = 0.0051) and misaligned (*M* = 0.9751, *SE*_M_ = 0.0050) different trials reaching similar performance levels. Nor was there an indication of an Alignment × Block interaction [$\hat{\varepsilon }$_GG_ = 0.96, *F*_(2.86, 77.33)_ = 0.79, *p* > 0.10, ${\hat{\eta }}_{p}^{2}$ < 0.05].

The correct response times showed clear evidence of a statistically significant composite effect [Alignment: *F*_(1, 27)_ = 19.54, *p* < 0.01, ${\hat{\eta }}_{p}^{2}$ = 0.42] after taking blocking effects into account [Block: $\hat{\varepsilon }$_GG_ = 0.69, *F*_(2.08, 56.15)_ = 22.91, *p* < 0.01, ${\hat{\eta }}_{p}^{2}$ = 0.46]. On average, subject responded to the aligned same trials (*mean* = 692, *SE*_M_ = 31) at a slower rate than on the misaligned same trials (*mean* = 658, *SE*_M_ = 27). There was no statistical evidence showing changes to the size of the composite effect throughout the experimental procedure [Alignment × Block: $\hat{\varepsilon }$_GG_ = 0.85, *F*_(2.56, 69.10)_ = 2.70, *p* > 0.05, ${\hat{\eta }}_{p}^{2}$ < 0.10]. Different trials, on the other hand, did not show any sign of an alignment effect [*F*_(1, 27)_ = 0.79, *p* > 0.10, ${\hat{\eta }}_{p}^{2}$ < 0.05] after controlling for general performance improvement [Block: $\hat{\varepsilon }$_GG_ = 0.62, *F*_(1.87, 50.55)_ = 9.25, *p* < 0.01, ${\hat{\eta }}_{p}^{2}$ = 0.26]. There was no significant Alignment × Block interaction [$\hat{\varepsilon }$_GG_ = 0.61, *F*_(1.83, 49.52)_ = 0.82, *p* > 0.10, ${\hat{\eta }}_{p}^{2}$ < 0.05].

Like the correct response times, strong evidence of a composite effect was found for the inverse efficiency data [Alignment: *F*_(1, 27)_ = 20.73, *p* < 0.01, ${\hat{\eta }}_{p}^{2}$ = 0.43] after taking blocking effects into account [Block: $\hat{\varepsilon }$_GG_ = 0.70, *F*_(2.09, 56.37)_ = 29.95, *p* < 0.01, ${\hat{\eta }}_{p}^{2}$ = 0.53]. On average, an efficiency difference of 45 ms/correct response between the aligned (*mean* = 720, *SE*_M_ = 35) and misaligned (*mean* = 676, *SE*_M_ = 28) same trials was observed. This effect remained stable throughout the experiment [Alignment × Block: $\hat{\varepsilon }$_GG_ = 0.91, *F*_(2.73, 73.67)_ = 1.48, *p* > 0.10, ${\hat{\eta }}_{p}^{2}$ < 0.05]. The different trials did not reveal a general alignment effect [Alignment: *F*_(1, 27)_ = 0.02, *p* > 0.10, ${\hat{\eta }}_{p}^{2}$ < 0.01]. Although a general performance increase was observed [Block: $\hat{\varepsilon }$_GG_ = 0.54, *F*_(1.62, 43.75)_ = 7.25, *p* < 0.01, ${\hat{\eta }}_{p}^{2}$ = 0.21], the alignment effect remained absent throughout all block stages [Alignment × Block: $\hat{\varepsilon }$_GG_ = 0.86, *F*_(2.59, 69.86)_ = 0.62, *p* > 0.10, ${\hat{\eta }}_{p}^{2}$ < 0.05].

**Figure 5:**
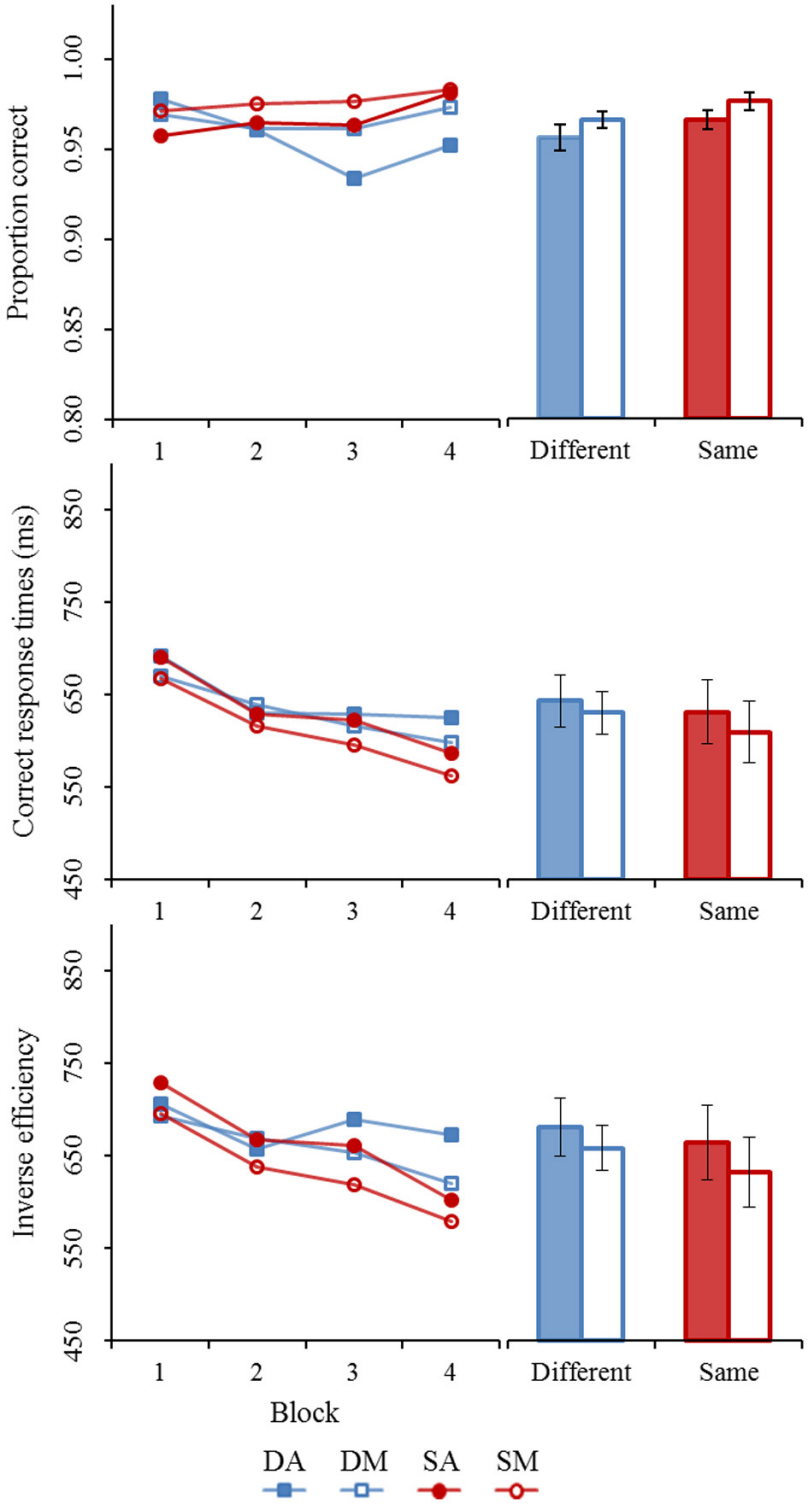
**Summary results of Experiment 2 (vertical body composite effect).** Each panel shows the averages for each block level (1–4) and for each trial type (different vs. similar). Averages across the blocks are also shown. Error bars represent standard error of the mean. (DA, different aligned; DM, different misaligned; SA, same aligned; SM, different misaligned).

Summarized results for Experiment 2 can be found in Figure 5. The proportion correct data of the same trials were analyzed first. Results indicated a significant alignment effect [Alignment: *F*_(1, 25)_ = 5.80, *p* < 0.05, ${\hat{\eta }}_{p}^{2}$ = 0.19]. This effect was consistent with a composite effect, with the aligned trials (*mean* = 0.9667, *SE*_M_ = 0.0052) having worse performance rates in comparison to the misaligned trials (*mean* = 0.9767, *SE*_M_ = 0.0049). This effect remained consistently present throughout the experiment [Alignment × Block: $\hat{\varepsilon }$_GG_ = 0.84, *F*_(2.53,63.33)_ = 0.42, *p* > 0.10, ${\hat{\eta }}_{p}^{2}$ < 0.05], despite a general performance increase over blocks [Block: $\hat{\varepsilon }$_GG_ =.082, *F*_(2.47,61.44)_ = 3.04, *p* < 0.05, ${\hat{\eta }}_{p}^{2}$ = 0.11]. Accuracy results for the different trials did not show a general alignment effect [Alignment: *F*_(1, 25)_ = 2.12, *p* > 0.10, ${\hat{\eta }}_{p}^{2}$ < 0.10] nor did accuracy for aligned vs. misaligned different trials deviate from each other throughout the experiment [Alignment × Block: $\hat{\varepsilon }$_GG_ = 0.93, *F*_(2.79, 69.81)_ = 0.98, *p* > 0.10, ${\hat{\eta }}_{p}^{2}$ < 0.05]. In fact, overall accuracy rates for the different trials remained roughly stable throughout the experiment [Block: $\hat{\varepsilon }$_GG_ = 0.79, *F*_(2.38, 59.57)_ = 1.35, *p* > 0.10, ${\hat{\eta }}_{p}^{2}$ < 0.05].

Next, correct response times were submitted to a repeated measures ANOVA. Results on the same trials indicated evidence of a clear alignment effect [*F*_(1, 25)_ = 12.94, *p* < 0.01, ${\hat{\eta }}_{p}^{2}$ = 0.34] after taking experimental progress into account [Block: $\hat{\varepsilon }$_GG_ = 0.69, *F*_(2.07, 51.85)_ = 13.85, *p* < 0.01, ${\hat{\eta }}_{p}^{2}$ = 0.36]. The alignment effect was in line with a composite effect revealing slower response rates for the aligned same trials (*mean* = 632, *SE*_M_ = 35) in comparison to the misaligned same trials (*mean* = 610, *SE*_M_ = 33). Furthermore, the composite effect remained consistent across the procedure [Alignment × Block: $\hat{\varepsilon }$_GG_ = 0.77, *F*_(2.32, 57.96)_ = 0.24, *p* > 0.10, ${\hat{\eta }}_{p}^{2}$ < 0.01]. The different trials showed a similar pattern as the accuracy data, with no sign of an alignment effect [*F*_(1, 25)_ = 2.35, *p* > 0.10, ${\hat{\eta }}_{p}^{2}$ < 0.10], a general performance improvement [Block: $\hat{\varepsilon }$_GG_ = 0.73, *F*_(2.19, 54.66)_ = 8.02, *p* < 0.01, ${\hat{\eta }}_{p}^{2}$ = 0.24] and no differential alignment effects were observed during the experiment [Alignment × Block: $\hat{\varepsilon }$_GG_ = 0.92, *F*_(2.76, 69.01)_ = 0.81, *p* > 0.10, ${\hat{\eta }}_{p}^{2}$ < 0.05].

The inverse efficiency data from the same trials showed a highly significant alignment effect [*F*_(1, 25)_ = 24.96, *p* < 0.01, ${\hat{\eta }}_{p}^{2}$ = 0.50] after controlling for a general blocking effect [Block: $\hat{\varepsilon }$_GG_ = 0.73, *F*_(2.18, 54.38)_ = 12.75, *p* < 0.01, ${\hat{\eta }}_{p}^{2}$ = 0.34]. Aligned same trials (*mean* = 665, *SE*_M_ = 40) were more efficiently solved in comparison to the misaligned same trials (*mean* = 633, *SE*_M_ = 38) providing evidence in line with a composite effect. This effect remained stable throughout the experimental procedure [Alignment × Block: $\hat{\varepsilon }$_GG_ = 0.79, *F*_(2.38, 59.51)_ = 0.31, *p* > 0.10, ${\hat{\eta }}_{p}^{2}$ < 0.01]. Different trials, on the other hand, showed no significant differences between the aligned and misaligned trials in general [Alignment: *F*_(1, 25)_ = 3.31, *p* > 0.05, ${\hat{\eta }}_{p}^{2}$ = 0.12] nor when taking block levels into account [Alignment × Block: $\hat{\varepsilon }$_GG_ = 0.82, *F*_(2.44,61.10)_ = 1.04, *p* > 0.10, ${\hat{\eta }}_{p}^{2}$ < 0.05]. Performance was generally stable throughout the experimental procedure [Block: $\hat{\varepsilon }$_GG_ = 0.88, *F*_(2.65, 66.27)_ = 2.67, *p* > 0.05, ${\hat{\eta }}_{p}^{2}$ < 0.10].

Finally, the composite effects found in Experiment 1 and Experiment 2 were compared by using independent *t*-tests on the alignment differences of the same trials. These tests showed no statistical differences between the two experiments in composite effect neither for the proportion correct data [*t*_(52)_ = 0.58, *p* > 0.10, ${\hat{\eta }}_{p}^{2}$ < 0.10], the correct response times [*t*_(52)_ = 1.24, *p* > 0.10, ${\hat{\eta }}_{p}^{2}$ < 0.10], nor for the inverse efficiency scores [*t*_(52)_ = 1.09, *p* > 0.10, ${\hat{\eta }}_{p}^{2}$ < 0.10].

## Discussion

The current paper presents evidence for holistic processing of neutral body postures. This evidence until now mainly comes from the body inversion effect (Reed et al., 2003; Minnebusch et al., 2009) and the part-whole effect (Seitz, 2002). The main purpose of the present study was to add to this evidence by studying the body composite effect. On each trial, participants were presented with two body postural configurations performed by the same figure and had to decide whether the predefined halves (top halves in Experiment 1 and right halves in Experiment 2) of the postures were the same or not, regardless of the task-irrelevant body part (bottom halves in Experiment 1 and left halves in Experiment 2) and we manipulated the alignment of the task-relevant and task-irrelevant body parts. We observed that, when the body parts were aligned, the task became more difficult. When a body can be seen in different spatial configurations (rather than only in a single, neutral, standing up posture), composite effects clearly can be observed, supporting the hypothesis that body postures, like faces, are processed in a holistic manner.

The currently available data on the body composite effect (Soria-Bauser et al., 2011; Robbins and Coltheart, 2012) are mixed. However, in these studies, the human body was always shown in a neutral standing-up posture and participants had to identify the figure rather than the posture. In analogy to faces which have been used in identity-based (e.g., Young et al., 1987; Palermo et al., 2011) but also in expression-based protocols (e.g., Calder and Jansen, 2005; Durand et al., 2007; Palermo et al., 2011), the primary aim of the current study was to study holistic processing of body postures while fixating the identity of the actor. As such, we used a posture-based approach instead of an identity-based approach. However, even within a posture-based approach, there are different means of operationalizations. For example, one way is to manipulate the amount of expressiveness present within the posture (e.g., van den Stock et al., 2008; de Gelder, 2012; Tanaka et al., 2012). Here, on the other hand, we opted for minimized amount of expressive and semantic information, in line with the original posture definition used by Reed et al. (2003), (2006), to bridge the research line of mere standing poses to emotionally neutral different posture configurations.

The current results are partially in line with the experimental findings of Robbins and Coltheart (2012). In their study, they tested top-bottom and left-right body integration by means of a within-subjects identity-based composite task. They found a subtle body composite effect smaller in size as compared to the face composite effect. Furthermore, their body composite effect pointed toward slightly more pronounced left-right integration as compared to top-bottom integration. Robbins and Coltheart (2012) suggested that this differential effect might be caused by disrupted head processing triggered by the vertical misalignment of the body halves. Even though they used masked heads, this explanation (although speculative) seems feasible in light of a coarse-to-fine framework of face processing (e.g., Rossion, 2013), in which holistic processing is present across different spatial resolutions, and thus might even occur for masked faces when they are part of a body configuration. However, the current study did not find any clear difference between the horizontal and vertical integration. One possible reason for this divergence in results might be laying within different choices in design.

Nevertheless, the current results combined with those of Robbins and Coltheart (2012), provide evidence for a body composite effect with both an identity-based and posture-based approach. These results are somewhat contradictory to the results of Soria-Bauser et al. (2011), who found no behavioral evidence of a body composite effect when using an identity-approach. Besides having different approaches to the composite effect, there are some differences in the experimental setup. For example, the stimuli used by Soria-Bauser et al. (2011) were about 3° by 3° of visual angle at a distance of 60 cm. One disadvantage might be that stimuli were presented on a scale so small that the task becomes much more difficult. When comparing their dimensions with our own (~15° at 58 cm) and those of Robbins and Coltheart (2012; ~16° at 60 cm), we might not directly compare results as the real-life equivalent distances are different. For example, when looking at two average sized men (say 1.80 m) at the dimensions given by Soria-Bauser et al. (2011), we would be comparing these persons from a distance of almost 35 m. In contrast, when using our own dimensions and those used by Robbins and Coltheart (2012), we would be seeing the same person at a distance almost 5 times closer (~6–7 m). Furthermore, Soria-Bauser et al. (2011) and Robbins and Coltheart (2012), compared their body composite effects with face composite effects. Again, when considering the real-life equivalents of their face stimuli, the face stimuli of Robbins and Coltheart should be seen at a distance around 85 cm, while those of Soria-Bauser et al. at a distance around 350 cm. As such, a direct comparison between studies, let alone across stimulus classes, should be made with extreme caution as the underlying real-life contexts vary widely in these studies.

In sum, we can conclude that, when a composite paradigm (e.g., in terms of ecological stimulus size), is optimized, a clear body composite effect can be documented in a theoretically motivated posture-based approach (instead of an identity-based approach). This suggests that human posture configuration are processed in a holistic way.

### Conflict of interest statement

The authors declare that the research was conducted in the absence of any commercial or financial relationships that could be construed as a potential conflict of interest.
